# Is the Pain Visual Analogue Scale Linear and Responsive to Change? An Exploration Using Rasch Analysis

**DOI:** 10.1371/journal.pone.0099485

**Published:** 2014-06-12

**Authors:** Paula Kersten, Peter J. White, Alan Tennant

**Affiliations:** 1 Person Centred Research Centre, School of Rehabilitation and Occupation Studies, Auckland University of Technology, Auckland, New Zealand; 2 Faculty of Health Sciences, University of Southampton, Southampton, United Kingdom; 3 Department of Rehabilitation Medicine, University of Leeds, Leeds, United Kingdom; Université catholique de Louvain, Belgium

## Abstract

**Objectives:**

Pain visual analogue scales (VAS) are commonly used in clinical trials and are often treated as an interval level scale without evidence that this is appropriate. This paper examines the internal construct validity and responsiveness of the pain VAS using Rasch analysis.

**Methods:**

Patients (n = 221, mean age 67, 58% female) with chronic stable joint pain (hip 40% or knee 60%) of mechanical origin waiting for joint replacement were included. Pain was scored on seven daily VASs. Rasch analysis was used to examine fit to the Rasch model. Responsiveness (Standardized Response Means, SRM) was examined on the raw ordinal data and the interval data generated from the Rasch analysis.

**Results:**

Baseline pain VAS scores fitted the Rasch model, although 15 aberrant cases impacted on unidimensionality. There was some local dependency between items but this did not significantly affect the person estimates of pain. Daily pain (item difficulty) was stable, suggesting that single measures can be used. Overall, the SRMs derived from ordinal data overestimated the true responsiveness by 59%. Changes over time at the lower and higher end of the scale were represented by large jumps in interval equivalent data points; in the middle of the scale the reverse was seen.

**Conclusions:**

The pain VAS is a valid tool for measuring pain at one point in time. However, the pain VAS does not behave linearly and SRMs vary along the trait of pain. Consequently, Minimum Clinically Important Differences using raw data, or change scores in general, are invalid as these will either under- or overestimate true change; raw pain VAS data should not be used as a primary outcome measure or to inform parametric-based Randomised Controlled Trial power calculations in research studies; and Rasch analysis should be used to convert ordinal data to interval data prior to data interpretation.

## Introduction

Visual analogue scales (VAS) are commonly used in clinical trials and other studies as primary [1]–[3] or secondary outcomes [4], [5] or as a tool to derive a health utility index [6]. The VAS is a 10 cm long straight line, marked at each end with labels which anchor the scale [7]. Vertical and horizontal presentations have been developed [8], although the horizontal version is the most common. In the context of pain, patients are asked to place a mark on the line at a point representing the severity of their pain where the anchors are ‘no pain’ and ‘pain as bad as it could be’ (labels vary between studies). Scores are noted in millimetres thus giving a total score range of 0–100 millimetres. Consequently, the VAS is often treated as an interval level scale (with equality between intervals [9]) and subjected to arithmetical operations (e.g. calculation of change scores) and parametric statistics. However, just because clinicians and researchers assume that the score in millimetres is interval in nature, this does not necessarily mean that patients score it as an interval scale. Indeed, some research suggests that patients find it difficult to judge how to rate their pain on the pain VAS line [10], [11], finding it ‘not very accurate’, ‘sort of random’, ‘almost guesswork’ or having to ‘work it into numbers first’ [10]. A study on business travellers also revealed that scores on a VAS (in this case 76mm in length) cluster into much smaller groups [12].

A wide range of Minimally (Clinically) Important Differences (MCID) in change scores on the pain VAS have been reported, ranging from nine to 30 millimetres in emergency departments [13]–[17]. Elsewhere changes of 33% [18] and 3.11 cm [19] have been shown as clinically meaningful post-operatively. However, others have shown that the pain VAS does not behave linearly for patients with all levels of pain [20]. For example, in a study of patients with extremity trauma, MCID for those with milder pain (less than 34 mm) was 13 (+/−14 SD) and 28 (+/−21) in those with scores of 67 mm or greater [20]. Similarly, in a study with patients experiencing subacute and chronic temporomandibular disorder pain, clinically important differences were dependent upon baseline pain [21]. In contrast, another study, specifically addressing the linearity of the pain VAS asked post-operative patients to consider their pain and score it on a VAS [22]. They were then asked to score the pain VAS again when they deemed that the amount of pain had halved. As pain halved similar changes in VAS scores were observed and the authors concluded that the scale was linear for those with mild to moderate pain. In addition, pain VAS measurement error has been reported as high as 9 mm [23] and 20 mm [24]. Consequently, change scores and the calculations of aspects such as MCID may be invalidated by the potential lack of interval scaling of the VAS, and further compromised by the magnitude of measurement error.

The Rasch measurement model, is ideally placed to examine whether a scale has internal construct validity, e.g. if the scale conforms to the definition of the construct [25] and, in this particular instance, whether or not it can be treated as an interval scale [26]. This is because where data are found to meet Rasch model expectations a transformation to interval scaling is obtained [27]. Consequently it becomes possible to compare the ‘raw’ (ordinal) score derived from the VAS with the transformed interval scale latent estimate of, for example, pain. Should the VAS be linear in its raw, ordinal score form there would be a linear association between it, and the interval scaled latent estimate. Recently, we have shown that the VAS scale, as used to measure the traits of ‘physical functioning’ and ‘pain on function’ in the Western Ontario and McMaster Universities Osteoarthritis Index (WOMAC), does not behave linearly and that it does not appear to be sensitive to change in the middle of the scale [28]. There is only one other paper that examined a VAS using Rasch analysis [29]. In this study, female patients with patellofemoral pain syndrome scored their pain on a VAS associated with each of 12 different activities (i.e. ‘pain on function’). Although the items were hierarchically ordered, it was found that patients did not use the VAS linearly over the full range and that the VAS could at best be considered to contain 10 category groupings. However, this was a small, underpowered study (n = 40) and made certain assumptions about the form of the Rasch model, which would be challenged in modern Rasch analysis protocols. Two other studies have employed the Rasch model to evaluate the VAS response format used in a clinical performance test [30] and a fatigue severity scale [31]. In both studies the VAS was converted into a 0–10 Likert scale, which makes assumptions about the scores within each 10 mm step on the scale. The results from these studies showed that categories needed to be combined to achieve fit to the Rasch model.

In summary, the VAS continues to be interpreted as an interval scale, rather than a categorical scale as proposed previously [11], [12], [29] and those studies that have used Rasch analysis have investigated scales that used the VAS format, rather than the pain VAS itself. This paper aims to examine the scaling properties and responsiveness of the pain Visual Analogue Scale using Rasch analysis and the implication of the findings for the interpretation of its sensitivity to change along the trait.

## Methods

Ethics approval for the study was gained from the Southampton & South West Hampshire and the Salisbury and South Wiltshire Research ethics Committees (approval number 170/03/t). Those eligible and willing to take part signed a consent form. Patients (n = 221, mean age 67, 58% female) were included if they had chronic stable pain predominantly from a single joint (hip or knee) of mechanical origin, were waiting for a hip (40%) or knee (60%) joint replacement, were not on active treatment (apart from their normal analgesia), and scored a minimum of 30 on the 100 mm VAS scale for pain on screening into the study. The latter criterion was included to ensure participants had at least moderate pain that might be helped with an intervention. Those with serious co-morbidity, pregnant, prolonged or current steroid use, or waiting for a joint revision were excluded.

Information was collected on a range of variables such as gender, age and the joint affected. Pain was measured at baseline by a VAS pain scale (once a day for seven days), then once a week for six weeks, and in the final week of the study pain was again measured once a day for seven days (follow-up point). Scores were recorded in a diary.

### Data analysis

A strategy was employed whereby the seven repeated VAS pain items across the baseline week, as described above, were treated as though they belonged to a single scale (and similarly for the seven daily measures at follow-up). In other words, the measurement for day one was considered item 1, for day two item 2, and so on. Since the thickness of a cross marked on a VAS may exceed one millimetre, or the interpretation of the exact location may vary by a millimetre, we divided the VAS scores by 2, thus reducing the range of each item to 0–50 points. We chose not to group the VAS data into 7–10 categories as proposed by some [11], [12], [29] because we specifically wanted to test if the raw data is indeed an interval scale.

Data from the items were fitted to the partial credit Rasch measurement model to determine if the ‘scale’ satisfied the expectation of the Rasch model [26], [32], in other words to examine fit to the model. The Rasch model is a probabilistic model, that expresses the probability of an item that represents a given level of ability (or as in our case level of pain) being passed (or agreed with) by people with a given level of ability (or pain), as a logistic function of the difference between item difficulty and person ability [26]. The Rasch model makes no distributional assumptions of the data under investigation. The unit of measurement in Rasch analysis is the logit (log odds probability units), which are interval based (e.g. the distance between each point on the scale is equal). Rasch analysis provides an integrated framework that evaluates if an outcome measure is internally valid and satisfies other requirements for constructing measurement, including the stochastic relationship between persons and items, as mentioned above, and assumptions of local independence, unidimensionality and invariance across groups. Each of these requirements will be explained in brief below.

Local independence: To achieve internal validity a scale must demonstrate local independence, in other words, responses to any given item should only depend on the trait level (in the case of pain VAS this would be how much pain someone has), and not on responses to previous items. The latter is called response local dependency [33]. With our repeated item design there was a risk that the response to one item (e.g. the VAS score for day 1) was dependent on the response to another item (e.g. the VAS score for day 5). Therefore, we gave particular emphasis at the outset to the formal test of local dependence. This was examined by examining the residual correlations between items, which should be no more than 0.20 above the average residual correlation [34]. Generally, where items are essentially replicates of existing items, as might be the case in the current design (and deliberately so) there might be an increase in reliability, and increased variance of person and item estimates [34], [35]. However, the primary goal of this analysis is to examine the scaling properties of the pain VAS, as opposed to validating a scale which has been artificially constructed for this purpose, and thus the concern is with the effect upon the latent estimate, which will be used for comparison with the raw VAS score.

Unidimensionality: The Rasch model requires the scale to measure one construct or dimension. This is examined by creating two subsets of items, which are identified by a principal component analysis of the item residuals, with those loading negatively forming one set and those positively loading the second set [36]. Strict unidimensionality is then examined using an independent t-test on the two estimates derived from the subtests for each respondent. If the 95% confidence interval of t-tests include 5%, unidimensionality is supported [36], [37].

Invariance: A scale will consist of items that are easier, and items that are harder to ‘achieve’ or ‘endorse’. It is important that this item ‘difficulty’ remains the same (is invariant) across different groups, such as age or gender. For example, we want to see that women and men provide the same response to an item when their overall level of experienced pain is the same. Similarly, responses should be invariant for other key factors such as joint affected. This is examined using analysis of variance of the residuals where the key group is the main factor. If variance is observed this is termed Differential Item Functioning (DIF). DIF can be uniform, i.e. bias is present consistently across the trait, or non-uniform (bias is not consistent across the trait) [38], [39]. Presence of DIF was examined for the person factors age groups, gender, practitioner, treatment group, consultation type, previous experience of acupuncture, or joint affected. It is also important that this hierarchical ordering of item difficulty remains stable across time thus giving confidence to the interpretation of the repeated measurement design. However, this has not been formally tested in the context of a pain VAS. This is of particular importance in the current study as the VAS data was derived from a repeated measurement design. Therefore, the analysis examined if item difficulty across days was stable (using DIF analysis). For this purpose we considered an estimated item difficulty range of 1 logit as stable [40], [41].

In addition to an examination of local independence, unidimensionality and invariance discussed above the Rasch analysis tests if item and person performances are as would be expected from the Rasch model. Thus, if the data fit the Rasch model (i.e. shows no deviation from the model expectations), a summary chi-square interaction statistic should be non-significant. Each item and person should also not deviate significantly from the Rasch model; this is explored by means of item and person fit residuals (which should be within the range of +/− 2.5), and the mean item and person residual fit statistics should be close to zero with a standard deviation of one, individual items should show non-significant chi-square fit statistics (Bonferroni adjusted).

In the case of the pain VAS we had divided scores by 2 and each item therefore had a range of 0–50, that is 51 categories. Thresholds are the points where the probabilities of a response of either 0 or 1, and 1 or 2 (and so forth) are equally likely. Log-transformed item scores generated from the response choices (categories) should reflect the increasing or decreasing latent trait to be measured. If the item responses options reflect increasing amount of experienced pain, then thresholds defining the categories should be ordered along the trait of pain likewise. When a given level of pain is not confirmed by the expected response option to an item, disordered thresholds will be observed. In such cases item categories should be grouped together until they are ordered.

Reliability was examined with a Cronbach alpha, deemed acceptable for group use if >0.7 [42]. Within the Rasch analysis reliability was also measured using the Person Separation Index [32], equivalent to alpha, but it can be calculated where missing values are present. Targeting of the scale (e.g. item locations) to the sample (e.g. person locations) was also explored.

Where a scale meets the expectations of the Rasch model (i.e. fit), the observed raw ordinal score gained through summation of the set of items can be transformed into interval scale measurement [32]. This interval scale is logit based. Consequently, this enabled us to examine responsiveness using both the observed, ordinal scores on the pain VAS, and those derived from the Rasch analysis (interval data). For ordinal data the mean baseline pain VAS scores were taken at baseline and follow-up (both recorded over a one week period). For the Rasch transformed (interval) scores, the person estimate at baseline and follow-up were used (and computed back from the logit scores to the same 0–50 sale). Standardised Response Means (SRM) were used to account for different levels of variance in the data at baseline and follow-up.

Bonferroni corrections were applied throughout the Rasch analysis to allow for multiple testing (P<0.01) [43]. Rasch analysis was conducted using RUMM2020 software [44]. Other analyses were carried out in SPSS15 [45] (descriptive statistics) and Excel 2003 (SRM's).

## Results

**Figure 1:**
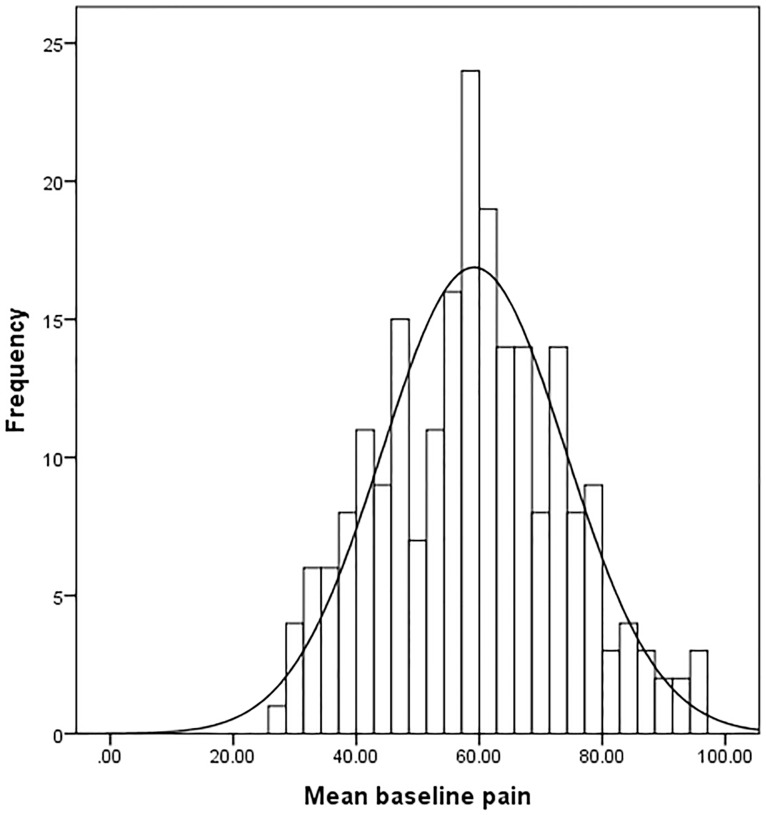
Data distribution baseline pain VAS scores (average over 7 days).

**Figure 2:**
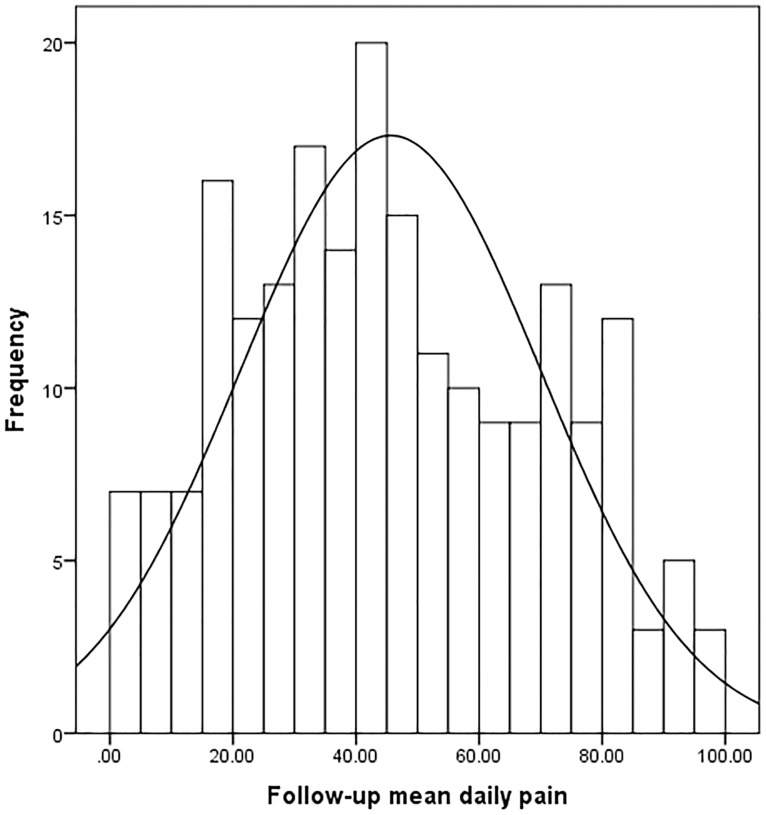
Data distribution follow-up pain VAS scores (average over 7 days).

Median pain scores over seven days were 59.4 mm at baseline and 42.7 mm at follow-up (table 1). Data followed a normal distribution at baseline and follow-up (table 1, figure 1 and 2). At baseline 70% of the scale was used and at follow-up this increased to 98%.

**Table 1:** Visual analogue scale distribution at baseline and follow-up (averaged over 7 days).

VAS scores (raw data)	Mean (SD)	Median	Interquartile range	Range*	Kurtosis	Skewness
Baseline	59.1 (14.9)	59.4	48.0 to 68.9	27.3 to 96.6	−0.382	0.133
Follow-up	45.6 (24.4)	42.7	26.8 to 67.0	0 to 98.4	−0.870	0.202

* The minimum baseline score is a little lower than 30 mm at screening, which took place a week before the commencement of the study.

### Fit to the Rasch model

The baseline pain VAS scores were tested against the Rasch model, which had a satisfactory fit (table 2, analysis 1) although the person fit residual standard deviation (SD) was high (1.7). All items had satisfactory fit statistics (non significant chi-squares and fit residuals between −2.5 and 2.5) and all thresholds were ordered. The PSI was high (0.90) and Cronbach alpha score was 0.88, but the scale demonstrated multi-dimensionality. There was no DIF by the person factors examined. Fifteen individuals did not fit the Rasch model (fit residuals outside the range of −2.5 and 2.5). Deleting misfitting cases (n = 15) resulted in a non-significant deviation from the Rasch model, including an acceptable level of the person residual standard deviation, and unidimensionality (table 2, analysis 2). This suggests that the lack of fit and unidimensionality was caused by some aberrant cases.

**Table 2:** Visual analogue scale scores fit to the Rasch model.

Analysis number	Item fit residual		Person fit residual		χ^2^ interaction		PSI	Unidimensionality Independent t-test (95% CI)
	Mean	SD	Mean	SD	Value (df)	P		
Baseline pain*								
1*	0.078	1.403	−0.775	1.714	21.72 (14)	0.084	0.90	8.1% (5.3 to 11.0)
2∞	0.020	1.128	−0.580	1.366	15.40 (14)	0.351	0.90	7.3% (4.3 to 10.3)
Post Pain								
3†	−3.304	2.190	−1.507	1.912	21.37 (14)	0.092	0.99	8.7% (5.6 to 11.7)
4∼	−0.849	0.755	−0.803	1.213	4.952 (6)	0.550	0.91	3.1% (0 to 6.1)

* Baseline pain: seven daily measures of pain on a VAS before the commencement of the trial, all cases included in the data set.

∞ Baseline pain: seven daily measures of pain on a VAS before the commencement of the trial, 15 misfitting cases have been excluded.

† Post pain contains seven daily measures of pain on a VAS after the completion of the trial, item threshold shave been anchored to baseline item thresholds, 15 misfitting cases have been excluded.

∼ Post pain contains three daily measures of pain on a VAS after the completion of the trial, item threshold shave been anchored to baseline item thresholds, 15 misfitting cases have been excluded and 4 items have been deleted.

**Figure 3:**
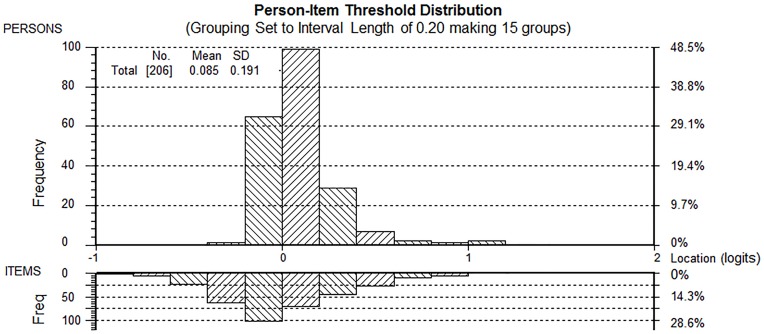
Person Item Threshold distribution (Pre VAS data). The graph displays the person-item threshold distribution map with the x-axes displaying location or difficulty of item thresholds (lower half) and location or level of pain reported on the VAS by participants (upper half). The y-axes display the frequencies of item thresholds (lower half) and participants (upper half). Thresholds of seven items are shown and it can be seen that the thresholds spread over 1½ logits only.

Item difficulty for the pain VAS was stable across the seven days (difficulty estimates were within a range of 0.07 logits, table 3). There were very few response categories on the VAS scales that were not used (figure 1). There were two sets of items (item 1&2, item 6&7) with residual correlations greater than 0.20 above the average residual correlation (i.e. 0.035). Each of these two sets of items were combined into a testlet (i.e. one testlet containing item 1&2, the other item 6&7) and tested against the Rasch model. Person estimates derived following this procedure did not differ significantly from those derived from analysis 2 (t-test, P = 0.687), and the PSI remained stable (0.89). The Person-Item threshold distribution map shows that participants are distributed in a similar fashion to the items (figure 3), which is indicative that the items measure pain along the construct from “no pain” to “worst imaginable pain”. It can also be seen that the item thresholds are closely located together, that is within 1½ logits of each other. However, as the mean error variance of the person estimates was very low (0.004) this does not affect the ability of the items to detect differences between groups of individuals with different levels of pain (PSI = 0.89).

**Table 3:** Visual analogue scale item difficulty (pre-data).

Item (day of completion)	Location (in logits)*
3	−0.023
7	−0.019
6	−0.009
4	−0.004
5	0.003
2	0.006
1	0.046

* The location represents the item difficulty in the Rasch model.

**Figure 4:**
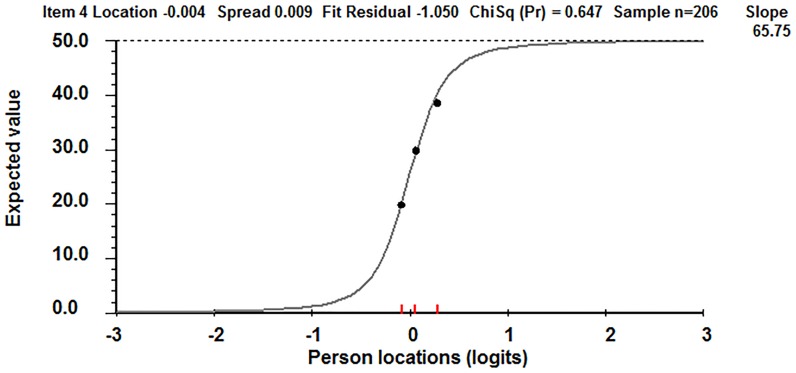
Item Response Curve for one VAS item. The Item Response Curve displays the expected raw score on the y-axis and the interval transformed log score on the x-axis.

The Item Response Curve of a typical VAS item in our dataset (figure 4), which plots patients' raw (ordinal) scores against the interval transformed scores, is very steep. As a single item, this shows clearly that the pain VAS works as an ordinal, non linear item.

Table 4 displays the pain VAS raw (ordinal) data points for the VAS measure on day 4 and converts these to interval scaling (note: as VAS scores were divided by 2 the scale ranges from 0–50 instead of 0–100 mm). As an example, on item 4 (day 4 VAS) a pain reduction of 10 mm (from 30 to 20) in the middle of the scale on the ordinal pain VAS line, converts to a change of 4 mm on the underlying interval scale. At the margins of the scale the reverse is true; small changes in the ordinal VAS score are linked to much larger interval equivalent changes. For example, the first 5 mm on the ordinal VAS line actually equals 13.5 mm on the interval scale.

**Table 4:** Vas conversion of raw (ordinal) data to interval data (item 4).

Raw Score (ordinal)*	Interval scores transformed back to 0–50 scale*	Raw Score (ordinal)*	Interval scores transformed back to 0–50 scale*
mm	mm	mm	Mm
			
0	0		
1	5.21	26	25.15
2	8.59	27	25.46
3	10.74	28	25.77
4	12.27	29	26.38
5	13.50	30	26.69
6	14.42	31	26.99
7	15.34	32	27.61
8	16.26	33	27.91
9	16.87	34	28.53
10	17.48	35	29.14
11	18.10	36	29.45
12	18.71	37	30.06
13	19.33	38	30.68
14	19.94	39	31.29
15	20.25	40	31.90
16	20.86	41	32.52
17	21.17	42	33.44
18	21.78	43	34.05
19	22.09	44	34.97
20	22.70	45	36.20
21	23.01	46	37.42
22	23.31	47	38.96
23	23.93	48	41.10
24	24.23	49	44.48
25	24.54	50	50.00

* The range is from 0 to 50 as VAS scores have been halved, thus scores range from 0–50.

obtain post pain VAS person estimates the post VAS items were anchored to threshold locations of the pre pain VAS data (to ensure calibration onto the same ruler). At follow-up the summary chi-square statistic was not significant (table 2, analysis 3). No items demonstrated DIF. However, item fit residuals were high and four items had unacceptable low negative fit residuals (<−2.5). These items were not locally dependent. Deleting these items resulted in a fit to the Rasch model (table 2, analysis 4). The PSI was high at 0.91, there was no local dependency and the item-thresholds were distributed over two logits. Cronbach alpha of follow-up items was 0.97. There was no DIF by time when pre and post data were stacked suggesting the pain VAS was invariant over time.

### Responsiveness

The SRM derived from the ordinal data overestimated the true responsiveness of the pain VAS by 59% (0.62 versus 0.39). The ordinal SRM (0.62) can be judged as medium to large in size, whereas the interval SRM (0.39) is small to medium [46].

**Figure 5:**
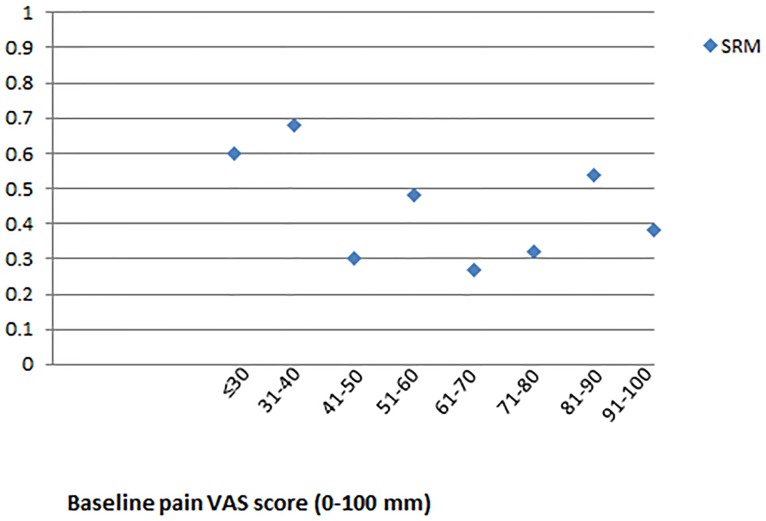
Standardised Response Means displayed by baseline raw pain VAS score. Inclusion criteria into the study included a minimum score on a single pain VAS of 30; although on the seven daily VAS measures some scored below 30 mm, these numbers were small. Standardised Response Means (SRM) data for those with daily measurs below 30 mm have therefore been combined into one group.

Further, as table 4 shows the pain VAS behaves in a more linear fashion in the middle of the scale, compared to the lower and upper end. We can therefore expect SRM's at the end of the scale to be larger than SRM's in the middle of the scale. To explore this, we divided the sample into groups as determined by their original baseline pain VAS scores (in mm, that is not halved) (e.g. those with baseline scores of 0–30 mm, 31–40 mm, 41–50 mm, 51–60 mm, 61–70 mm, 71–80 mm, 81–90 mm, 91–100 mm) and SRM's were calculated for each of these groups using interval data. Figure 5 clearly shows that SRM's medium to large (≥0.50) in the groups that started off with a lower pain VAS (range 0–40 mm); they then fall to small-medium (≥0.20 and <0.50) in groups with more moderate baseline scores (41–80 mm); the SRM is medium to large for those people with higher (81–90 mm) baseline scores. These findings confirm that the pain VAS does not behave in a linear fashion. This has consequences for the commonly reported MCID of 13 mm, since 13 mm change on the ordinal pain VAS represents fewer interval points in the middle of the scale than on the margins. Thus, the MCID is not stable across the construct of pain when measured with the pain VAS.

## Discussion

For Rasch analyses, reasonably well targeted samples of 150 are reported to have 99% confidence that the estimated item difficulty is within +/− ½ logit of its stable value [40], [41]. Our ‘reasonably targeted’ sample of 206 was therefore deemed adequate for the purpose of this analysis and our study is the first with sufficient power to explore the internal validity of the pain VAS using Rasch analysis. We paid particular attention to the assessment of local dependency and imposed a stringent criterion (i.e. residual correlations between items should be no more than 0.20 above the average residual correlation [47]). Thus, we specifically tested if local dependency affects person estimates and found that this was not the case.

Rasch analysis allows an investigation of person fit. Essentially this examines if people use the scale as expected, given the item difficulties and their total scores on the scale. In traditional psychometric testing this is not examined; indeed, the assumption is made that people respond to items in the way intended. From the fit statistics we cannot determine with certainty why 7% of our participants did not fit the Rasch model. It could be that they found the VAS scale difficult to understand and score; a qualitative investigation alongside this quantitative analysis could shed light on this. Taking these people out of the remaining analysis was important as their data led us to think the scale was not unidimensional; this would have been an incorrect conclusion as shown above.

Three key findings arise from our study, which advance the field of research on the pain VAS. Firstly, our study showed that item difficulty of the pain VAS remained stable over a one-week period. This suggests the Pain VAS is interpreted in the same manner, irrespective of when it is completed and even when patients can see their previous scores. This lends support for the internal validity of the pain VAS. Secondly, we found that the pain VAS data fit the strict Rasch model, indicating it has internal validity. Thirdly, and importantly, the present analysis shows clearly that the pain VAS is an ordinal scale with a number of problems which makes its interpretation less straight forward:

The pain VAS thresholds spread only over 1½ to two logits. Such findings could occur if the sample is overly homogeneous. However, this was not the case here as table 1 and figures 1 and 2 showed that the narrow range occurred despite the use of 70% of the scale at baseline and 98% of the scale at follow-up. Thus, the narrow range of thresholds is due to the lack of sensitivity of the VAS pain scale to distinguish between groups of people with different levels of pain. This finding is in contrast to commonly held beliefs that the VAS is sensitive in measuring pain. The range of logits found here is similar to the findings in the earlier WOMAC VAS scale study [28].

Change in scores at the margins of the pain VAS, while gaining few raw score points, reflects considerable metric change. By contrast, moving across the middle of the pain VAS, gaining many raw score points, reflects little change on the metric. It follows from this that the magnitude of SRM's depended on baseline pain VAS scores. For those with initial scores at the upper end or the lower end of the scale the SRMs were substantially higher on the metric than the ordinal equivalent. The pain VAS could therefore be said to be sensitive to change for those groups of patients. However, SRMs on the metric for those patients with more moderate pain (i.e. in the middle of the scale) were low and responsiveness for this group of patients is therefore poorer. The variable SRMs that we found lend support to the findings by others [20], [21], though these studies used parametric statistics. The fallibility of using parametric statistics on the VAS was clearly demonstrated in our analysis which provided evidence that the pain VAS does not behave in a linear fashion despite its large number of categories.

These findings challenge the interpretation of pain VAS change scores as reported in the literature [4], [48], [49]. In a clinical trial comparing two different techniques of high tibial osteotomy patients' pain VAS scores changed on average 23 mm (lateral closing wedge technique) and 27 mm (medial opening wedge technique) [4]. These changes were not statistically significant. Perhaps this is not surprising as when we converted their ordinal pain VAS change scores to interval change scores, using our Rasch data, the change scores were only 7 mm and 8 mm respectively. Interestingly, both groups had baseline scores (59 mm vs 63 mm), which lie in the band of small to medium SRM's as found in our study (figure 5). Similarly, in a trial comparing acupuncture to placebo needling for the treatment of acute low back pain, patients scored their average and their worst pain on a VAS [49]. Average baseline pain VAS scores were 56.2 mm in the group that received verum acupuncture and 62.6 mm in the group that received sham acupuncture. Although pain VAS scores improved with 28.9 mm and 26.3 mm respectively (larger than the widely reported 13 mm MCID) this was not statistically significant. Again, those ordinal changes are overestimated as when using the Rasch transformation, these converted to 9 mm and 8 mm respectively. Changes in the worst pain VAS scores were significant for the verum acupuncture group at follow-up (a 43 mm change, or converted to interval data 17 mm change).

There are some limitations to the study. The current study included patients with osteoarthritis who were waiting for a joint replacement and further research needs to explore the pain VAS in other populations. In addition, we did not ask participants if they judged the change in their pain VAS scores as clinically meaningful since this was not the primary aim of the study. However, we are able to make some judgements on Minimally Clinically Important Differences (MCID) since we demonstrated the variability in 13 mm raw change scores (a commonly reported MCID) once transformed to interval data.

## Conclusions

In conclusion, we have established that repeated pain VAS data meets the strict requirements of the Rasch model, including unidimensionality, and that it is internally valid. Thus, the pain VAS is a valid tool for measuring pain at one point in time. However, the study has provided strong evidence that the pain VAS does not behave linearly and that as a consequence, SRMs will vary along the trait of pain. The contention that the pain VAS is a ratio scale for pain measurement is therefore not valid [50]. Thus, Minimum Clinically Important Differences using raw data, or change scores in general, are meaningless, as these will either under- or overestimate true change. Our findings highlight the necessity to use Rasch analysis to convert ordinal data to interval data prior to interpretation and build on our recent review of the VAS [51]. More importantly, our findings raise serious issues for researchers in that raw pain VAS data cannot be used in power calculations based upon interval scaled parametric assumptions. If a raw pain VAS is used as a primary outcome measure, it must either be subjected to non-parametric statistics, or transformed by Rasch analysis into an interval scale latent estimate where such statistics can be used, given appropriate distributional assumptions are met.
